# Privacy Concerns Over the Use of Webcams in Online Medical Education During the COVID-19 Pandemic

**DOI:** 10.7759/cureus.13536

**Published:** 2021-02-24

**Authors:** Mohammad H Rajab, Mohammed Soheib

**Affiliations:** 1 Epidemiology and Public Health, Alfaisal University College of Medicine, Riyadh, SAU; 2 Medicine, Alfaisal University College of Medicine, Riyadh, SAU

**Keywords:** webcam, alfaisal university, online medical education, covid-19 pandemic

## Abstract

The emergence of the coronavirus diseases 2019 (COVID-19) pandemic has transformed traditional classroom education to online learning worldwide. Challenges to this sudden transformation include protecting student privacy while using online medical education services. Consequently, dealing with challenges to online medical education became a heated debate at Alfaisal University. This study aimed to determine the challenges of using webcams during online classes and the ensuing implications for medical education during the COVID-19 pandemic. A cross-sectional study was conducted during the 2020-2021 academic year. The study investigators developed and validated a self-administered online questionnaire that targeted preclinical and clinical medical students at Alfaisal University, which is a private not-for-profit academic institution that was founded in 2008. Alfaisal University consists of five colleges: Medicine, Business, Engineering, Pharmacy, and Science. A total of 319 medical students completed the study questionnaire, with a response rate of 25%. The median age of the study sample was 21 years, with 63.3% females; 78.1% were in preclinical (first to third) years and 21.9% were in clinical (fourth to fifth) years. Of the respondents, 76.5% reported not using webcams when communicating via social media and 64.3% preferred blended learning, i.e., a combination of face-to-face and online learning. An overwhelming majority (91.5%) of study respondents were against using webcams in online classes for reasons including privacy (88.4%) and anxiety (64.4%). Privacy was the main concern of study respondents. Information about online privacy, including information being collected and how it will be used, should be provided to the students. Effective strategies to improve online learning experiences and protect the privacy of students should be explored.

## Introduction

On March 11, 2020, the coronavirus outbreak was deemed as a pandemic by the World Health Organization (WHO) [1]. A pandemic, as defined by the WHO, is “the worldwide spread of a new disease” [2]. The coronavirus pandemic suddenly forced faculty and students to rely more on technology [3].

During the coronavirus diseases 2019 (COVID-19) pandemic, universities around the globe were obliged to abruptly consider online content delivery modes on a massive scale [4-6]. In light of the COVID-19 pandemic, video conferencing using web services such as Zoom® and Microsoft Teams® have gained popularity among higher education institutions [7,8]. However, protecting student privacy while using webcams became a challenge. Consequently, dealing with challenges to online medical education became a heated student-faculty debate at Alfaisal University [9].

Prior studies indicate that students have had mixed experiences with video conferences [10]. Video conferencing has been widely employed as an educational tool in televising surgical procedure skills and has largely yielded positive feedback from medical students [11]. In exploring the factors that influence student satisfaction, Dawson identified that students felt that the quality of interactions during the session and social presence were the main factors that contributed to students’ satisfaction with video conferencing [12]. In a similar study involving video conference sessions, student participation was a stronger predictor of student exam performance than the use of different tools and technologies [13]. Another study evaluated the use of video conferencing among team members working on a research project; the participants attributed webcam use to the development of an increased sense of belonging and social connection among themselves [14].

Despite its benefits, video conferencing - with a webcam - has many drawbacks and challenges in its adoption. Doggett and Doggett reported that over half of the participants in their study felt that technical issues during video conferencing hindered the communication between the students and instructors [15]. This is also supported by evidence that showed that transmission delays negatively affected the perception of the participants towards the speaker, leading to a negative misattribution of the delay to the speaker’s behavior and character [16]. Furthermore, using a webcam may hinder students’ and instructors' focus on the session as they become more aware of their online presence [17].

From the students’ perspective, the main concerns with using webcams are due to issues with privacy and the feeling of unease of having their face broadcast during the session. Other reasons for not using a webcam were shyness, discomfort, and sometimes fatigue [18,19]. In a large-scale study, which surveyed students on their webcam usage in educational sessions across different faculties, only about 7% of students reported using their webcam on all occasions [20]. The reasons cited by students - those who did not use a webcam - were privacy concerns, technical issues, and just feeling uncomfortable [18,21]. Among experienced learners and instructors, webcam adoption is higher at the beginning of the course; however, it falls by the end of the course. Students who chose not to use webcams did so more often due to privacy reasons than due to technical issues [18].

Medical education is unique, as it is primarily divided into preclinical and clinical phases that differ in their teaching style. At the College of Medicine at Alfaisal University, the undergraduate medical program consists of two phases - the first three years make up the basic science or preclinical phase (years 1-3) followed by two years of clerkships, which constitute the clinical phase (years 4 and 5). Clinical education focuses on the development of the students’ emotional intelligence and social competencies throughout their professional careers [22]. It has been stated that much of the education in clinical medicine occurs outside the formal learning environment, which includes the imparting of values and the cultivation of professionalism through interaction between students, residents, and attendings [23].

In theory, video conferencing using a webcam could provide an alternative that closely emulates a classroom environment [20]. However, insights into the challenges with the webcam - its use or non-use among medical students in different phases - would equip medical schools to develop optimal policies and guidelines to provide effective synchronous learning sessions during a pandemic. The aim of this study was to determine the challenges faced by medical students related to the use of webcams in live online classes and implications for medical education during a pandemic.

## Materials and methods

To achieve the study aim, we conducted a descriptive cross-sectional study College of Medicine at Alfaisal University in Saudi Arabia during the 2020-2021 academic year. Alfaisal is a private not-for-profit academic institution that was founded in 2008. It consists of five colleges: Medicine, Business, Engineering, Pharmacy, and Science. The study population included preclinical and clinical medical students of Alfaisal University. The study was approved by the Institutional Review Board of Alfaisal University.

The study investigators developed a self-administered online questionnaire using Google Forms® to investigate the use of webcams in live online classes during the ongoing COVID-19 pandemic. The medical students were informed via email or social media university groups about this survey and were later reminded to complete the questionnaire.

The study questionnaire contained an introductory paragraph that informed participants of the study aim, the confidentiality of their responses, and the freedom not to participate. The confidentiality of survey responses was maintained throughout the study and subsequently. The study questionnaire contained a combination of closed- and open-ended questions. The closed-ended questions included inquiries into participants’ demographics and experience with webcam use during the pandemic. Open-ended questions included solicitations of recommendations. As part of the questionnaire development process, five faculty members were asked to pilot-test the survey draft. The pilot data were used for survey validation only and were excluded from the final data analysis.

To start the data collection phase, an introductory email was sent to members of the study population. The introductory email informed the medical students of the study aim and solicited their participation. Next, the survey’s weblink was sent to the study population using the Institutional Email System. Subsequently, two follow-up email reminders were sent to the same group. Students who have already graduated, faculty, and staff were excluded from participating in the study.

The total number of the study population, i.e., medical students at Alfaisal University, was close to 1,300. As we chose a margin of error of 5% and a confidence level of 95% for our study, the study sample size was a minimum of 300 respondents. Categorical variables including gender, phase, marital status, and living status were reported as numbers and percentages, and age was reported as median and range. Data management and analysis were performed using the Jamovi Software, Version 1.2.27 for Windows.

## Results

A total of 319 preclinical and clinical medical students responded to the survey. The response rate was approximately 25%. We excluded the questionnaires of 10 students from the analysis since their responses were incomplete. The median age of the study sample was 21 years (range: 17 to 28 years). Of the respondents, 63.3% were females, 78.1% were in preclinical (1-3) years, 21.9% were in clinical (4, 5) years, 80.6% were living with family, 9.4% were living with a roommate, and 9.1% were living alone. The characteristics of the study respondents are presented in Table 1.

**Table 1:** Characteristics of the study respondents

	Participants (N=319)
Age	
Median (range), years	21 (17-28)
Gender	
Female, N (%)	202 (63.3)
Male, N (%)	117 (36.7)
Phase	
Preclinical (years 1-3), N (%)	249 (78.1)
Clinical (years 4 and 5), N (%)	70 (21.9)
Marital status	
Single, N (%)	309 (96.9)
Married, N (%)	5 (1.6)
Other, N (%)	5 (1.6)
Living status	
Alone, N (%)	29 (9.1)
With family, N (%)	257 (80.6)
With a roommate, N (%)	30 (9.4)
Other, N (%)	3 (0.9)

Of the respondents, 64.3% preferred blended learning, 21.3% preferred traditional face-to-face instructions, and only 14.4% preferred online instruction. The majority (76.5%) of respondents reported not using cameras when communicating via social media. The overwhelming majority (91.5%) of study respondents preferred not using cameras during online classes. Participants’ preferences during the COVID-19 pandemic are presented in Table 2.

**Table 2:** Students online preferences during the COVID-19 pandemic

	Participants (N=319)
Preference	
Blended learning, N (%)	205 (64.3)
Face-to-face, N (%)	68 (21.3)
Online only, N (%)	46 (14.4)
Camera use with social media	
No, N (%)	244 (76.5)
Yes, N (%)	75 (23.5)
Camera use in online classes	
No, N (%)	292 (91.5)
Yes, N (%)	27 (8.5)

The reported webcam use challenges during online lectures at this institution were mainly privacy (88.4%), anxiety (64.4%), and stress and fatigue (50%). The reported webcam use challenges during the COVID-19 pandemic are presented in Table 3.

**Table 3:** Reasons for not using webcams in online medical education activities

	Participants (N=319)
Privacy	
No, N (%)	34 (11.6)
Yes, N (%)	258 (88.4)
Anxiety	
No, N (%)	104 (35.6)
Yes, N (%)	188 (64.4)
Stress and fatigue	
No, N (%)	146 (50.0)
Yes, N (%)	146 (50.0)
COVID-19 pandemic related	
No, N (%)	260 (89)
Yes, N (%)	32 (11)
Lack of technology	
No, N (%)	268 (91.8)
Yes, N (%)	24 (8.2)
Technical issues	
No, N (%)	198 (67.8)
Yes, N (%)	94 (32.2)

In this study, only 27 (8.5%) students preferred using a webcam during the educational sessions. The reported reasons for using webcams during the educational sessions included social engagement (77.8%), accountability (85.2%), and mimicking classrooms (55.6%). A summary of the reported reasons for using webcams is presented in Table 4.

**Table 4:** Reasons for using webcams in online medical education activities

	Participants Who Used Webcams (N=27)
Social engagement during the pandemic	
No, N (%)	6 (22.2)
Yes, N (%)	21 (77.8)
Provides a sense of accountability	
No, N (%)	4 (14.8)
Yes, N (%)	23 (85.2)
Mimics traditional classroom setting	
No, N (%)	12 (44.4)
Yes, N (%)	15 (55.6)

Preferences of learning modes by gender revealed that 17.8% of female students reported a preference for face-to-face learning compared to 27.4% of male students who reported the same preference. Preference for blended learning by gender revealed that 72.8% of female students preferred blended-learning compared to 49.6% of male students. Also, 9.4% of female students preferred the online-only learning mode compared to 23.1.4% of male students. A summary of the preference of learning mode by gender is presented in Table 5.

**Table 5:** Preference of learning mode by gender

	Female, N (%)	Male, N (%)
Learning mode		
Blended learning, N (%)	147 (72.8)	58 (49.6)
Face-to-face, N (%)	36 (17.8)	32 (27.4)
Online only, N (%)	19 (9.4)	27 (23.1)

## Discussion

Since the beginning of the pandemic, medical education unexpectedly shifted to online teaching [24]. As a result, medical schools did not have sufficient time to properly prepare for this sudden shift in how instructions are delivered. One of the main questions that faculty and administrators faced at our institution was whether students were required to use a webcam during online classes.

Webcams are video cameras embedded in most new laptops or placed on a desk used in online communication. Switching to online learning and education due to the advent of the COVID-19 pandemic prompted many schools around the world, including Alfaisal University, to use webcams with applications such as Zoom. The Zoom software, which has gained popularity worldwide during the COVID-19 pandemic, is known to be vulnerable to cyber attacks [25]. In this study, almost all respondents preferred not to use a webcam during live online medical education activities mainly to maintain privacy.

The introduction of software programs such as Zoom and Microsoft Teams has highlighted many concerns for privacy and data security. One of the privacy concerns has been the many instances of “Zoom-bombing”, an instance where a person with ill-intent, most often a stranger, joins the Zoom session and disrupts the session by use of expletives or abusive language [26]. This is not just limited to Zoom but also other video-conferencing programs that provide similar services. Other more subtle and concerning issues for privacy have been programs’ tracking and data collection policies. Some of these programs collect and use tracking data from the meetings, sometimes without the participants being aware of it, and use this data for providing personalized advertising to users. The surge in the usage of these programs during the COVID-19 pandemic increased the level of concern [27]. In our study, the majority of respondents reported not using a webcam with social media.

In the United States, many states have passed laws to protect student privacy [25]. Still, many students participating in online education in other countries around the world are vulnerable. Furthermore, governments may not require that schools provide students with information on how their data are used [25]. Therefore, there is a need to provide students with information regarding their online privacy. At the same time, more regulations protecting student online privacy must be enacted.

Our results on students’ lack of preference for webcam align with a similar study conducted in a European university setting [20]. Additionally, in the same European study, the students’ main concern with webcam usage was privacy, and this finding also aligns with the findings of our study [20]. In our study, anxiety was the second most important reason for not using webcams in online classes. In a previous study on the same population, anxiety was one of the main challenges to online medical education [5].

The majority of the respondents in our study preferred blended learning, i.e., a combination of face-to-face and online learning, as reported in a previous study [5]. Blended learning is becoming more accepted by academic communities because it combines the best of both worlds [28]. Using a webcam requires faster internet connections and newer computers, and some students may not be able to afford new technology [29]. However, lack of technology and technical issues were not among the main reasons that prevented study respondents from using webcams.

Reasons for using webcam included social engagement during the isolation period of the pandemic. Using a webcam during live sessions mimics face-to-face group meetings so that students feel like part of a community [29]. Another reason for using webcams that study respondents reported was to provide a sense of accountability.

Gender played a role in this study. The majority (63.4%) of the study respondents were females, which could potentially be due to elevated privacy concerns among female students. Among students who preferred blended learning, the percentage of female students was higher than the males. However, among students who do not prefer webcam use in online medical education, the percentages of female and male students were similar.

In this study, participants were given the option to comment on the use of webcams for online sessions based on their experiences during the past few months. Many of the comments were reiterating students' preference for not using webcams. Students reported that they experienced increased stress and anxiety while having their cameras turned on because of concerns about their privacy, looks, and family members in the background. Furthermore, many believed that policies that would require the use of cameras during the online session would be detrimental to their learning experience. This feedback from the students underpinned our study and prior studies’ findings that privacy was the chief concern among students when it came to using webcams [18,20].

Many students also mentioned that they did not view the use of a webcam as practically useful for lectures with a large group of students. Students recognize the differences between the large group activities and small group activities and assert that there are different roles for webcams in each group. One student mentioned that it seemed much more “relevant” and “easier” for students to have their cameras turned on in smaller sessions such as problem-based learning sessions and small group discussions, owing to more interactions and exchange of information between students and instructors during these sessions. Many believed that having to use a camera in large groups interfered with their ability to pay attention during class.

Nevertheless, while the majority considered the use of webcams as distracting, a small minority of students believed that webcams helped make the session “feels like an actual class” and believed it should be required for everyone. These students alluded to a lack of active participation in online sessions as an incentive for having their cameras turned on. Many of the respondents requested the optional use of webcams in online classes.

Study respondents believed that the focus on webcams for online sessions is misplaced. The larger challenge is that the faculty and students are not given sufficient training for online classes. Students believed that this was a more crucial factor that explained the differences between online sessions during the pandemic and traditional classes. This highlights the importance of proper training and continued support to the faculty in helping them deliver content to the medical students. Hence, proper training to adapt to online sessions can contribute to student satisfaction with online courses.

This cross-sectional study has limitations attributable to constraints on research design or methodology. These limitations may have impacted the study findings. The main limitations were non-response bias and the low response rates. A potential reason for such limitation was conducting the study close to the final exams. In addition, most of the preclinical participants were from the second and third years. The participation of the first-year medical students was minimal. Another limitation was the paucity of published related research. Finally, this study included only medical students. The presence of these limitations lessens the generalizability of the study results [30]. Despite these limitations, our study provides relevant information about medical students’ concerns over the use of webcams in online medical education during the COVID-19 pandemic. Such information provides a foundation for in-depth research studies.

## Conclusions

The majority of respondents preferred not to use webcams during online academic activities for reasons including privacy, anxiety, stress, and fatigue. The respondents believed that webcams are distractions that can diminish students' attention, pressurize them, increase their anxiety, and potentially expose their home environments. The students felt that the value provided by using webcams in online lecture settings is minimal, particularly when weighed against their privacy concerns. Moreover, issues with online lectures can be dealt with effectively without the use of webcams.

Academic medical centers should provide information about students’ online privacy including what information is being collected and how it will be used. Plans to protect online privacy must be designed taking into consideration students’ feedback. Effective strategies to improve the online learning experiences without the mandatory use of webcams should be explored. These strategies include providing sufficient training and support for faculty and students.
